# Therapeutic Effects of Atranorin towards the Proliferation of *Babesia* and *Theileria* Parasites

**DOI:** 10.3390/pathogens9020127

**Published:** 2020-02-17

**Authors:** Amany Magdy Beshbishy, Gaber El-Saber Batiha, Luay Alkazmi, Eman Nadwa, Eman Rashwan, Ahmed Abdeen, Naoaki Yokoyama, Ikuo Igarashi

**Affiliations:** 1 National Research Center for Protozoan Diseases, Obihiro University of Agriculture and Veterinary Medicine, Nishi 2-13, Inada-cho 080-8555, Obihiro, Hokkaido, Japan; amanimagdi2008@gmail.com (A.M.B.); yokoyama@obihiro.ac.jp (N.Y.); igarcpmi@obihiro.ac.jp (I.I.); 2 Department of Pharmacology and Therapeutics, Faculty of Veterinary Medicine, Damanhour University, Damanhour 22511, Al Beheira, Egypt; 3 Biology Department, Faculty of Applied Sciences, Umm Al-Qura University, Makkah 21955, Saudi Arabia; lmalkazmi@uqu.edu.sa; 4 Department of Pharmacology and Therapeutics, College of Medicine, Jouf University, Sakaka 72345, Saudi Arabia; emanhassannadwa@yahoo.co.uk; 5 Department of Medical Pharmacology, Faculty of Medicine, Cairo University, Giza 12613, Egypt; 6 Department of Physiology, College of Medicine, Al-Azhar University, Assuit 71524, Egypt; dremanrashwan2020@gmail.com; 7 Department of Physiology, College of Medicine, Jouf University, Sakaka 42421, Saudi Arabia; 8 Department of Forensic Medicine and Toxicology, Faculty of Veterinary Medicine, Benha University, Toukh 13736, Egypt; ahmed.abdeen@fvtm.bu.edu.eg

**Keywords:** atranorin, drug discovery, piroplasmosis control, chemoprophylactic agents, *Babesia* sp., *Theileria* sp.

## Abstract

Atranorin (ATR), is a compound with multidirectional biological activity under different in vitro and in vivo conditions and it is effective as an antibacterial, antiviral, antiprotozoal and anti-inflammatory agent. In the current study, the in vitro as well as in vivo chemotherapeutic effect of ATR as well as its combined efficacy with the existing antibabesial drugs (diminazene aceturate (DA), atovaquone (AV) and clofazimine (CF)) were investigated on six species of piroplasm parasites. ATR suppressed *B. bovis, B. bigemina, B. divergens, B. caballi* and *T. equi* multiplication in vitro with IC_50_ values of 98.4 ± 4.2, 64.5 ± 3.9, 45.2 ± 5.9, 46.6 ± 2.5, and 71.3 ± 2.7 µM, respectively. The CCK test was used to examine ATR’s cytotoxicity and adverse effects on different animal and human cell lines, the main hosts of piroplasm parasites and it showed that ATR affected human foreskin fibroblasts (HFF), mouse embryonic fibroblast (NIH/3T3) and Madin-Darby Bovine Kidney (MDBK) cell viability in a dose-related effect with a moderate selective index. The combined efficacy of ATR with DA, CF, and AV exhibited a synergistic and additive efficacy toward all tested species. In the in vivo experiment, ATR prohibited *B. microti* multiplication in mice by 68.17%. The ATR-DA and ATR-AV combination chemotherapies were more potent than ATR monotherapy. These results indicate the prospects of ATR as a drug candidate for piroplasmosis treatment.

## 1. Introduction

Currently, chemotherapy treatments against piroplasmosis remain inadequate. Oxytetracycline, imidocarb dipropionate and diminazene aceturate (DA) are considered the main choices for the treatment of piroplasm parasites that infect cattle and horses, although there have been cases of toxic effects related to these drugs [1,2], as well as the development of imidocarb dipropionate–resistant *Theileria equi* and DA-resistant *Babesia gibsoni* [3,4]. Combined atovaquone (AV) and azithromycin therapy is still the best choice against human zoonotic babesiosis due to its low side effects [5]. In order to address the shortcomings in the therapeutic options available for these parasitic diseases, new drugs are required [2].

**Figure 1:**
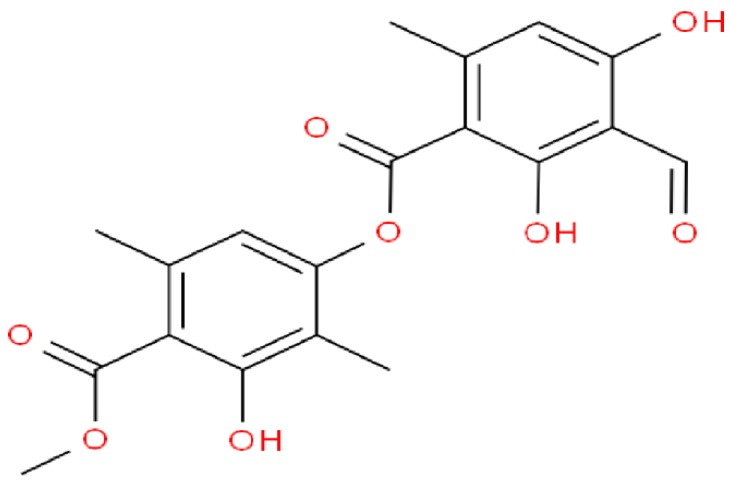
The chemical structure of atranorin.

Atranorin (ATR; C_19_H_18_O_8_; Figure 1), is one of the β-orcinol derivatives that are often found in various lichen families, including *Parmeliaceae*, *Cladoniaceae*, *Lecanoraceae*, and *Streocaulaceae*. It was first isolated by Hesse in 1898 and since then its pharmacological and biological properties have been extensively examined and evaluated [6]. Notably, previous studies documented its in vitro as well as in vivo activities as an anti-inflammatory, antioxidant, analgesic, immunomodulatory, anticancer, antiprotozoal, antiviral, antifungal, and antibacterial agent against Gram-negative bacteria [7,8,9,10]. Ranković et al. [11] documented the potent antibacterial activity of ATR against the most dangerous respiratory tract bacteria, *Staphylococcus aureus*, comparable to streptomycin. Moreover, Vu et al. [12] revealed that ATR possesses antiviral activity against the hepatitis C virus (HCV) with an IC_50_ value equal to 22.3 ± 8.0 µM. ATR also reportedly possesses antimalarial activity toward the chloroquine-resistant W-2 and the CAM10 and SHF4 *Plasmodium falciparum* isolates [13,14]. Experiments on animals revealed the antioxidant as well as anti-inflammatory potentials of ATR which indicate that it might be an important therapeutic component in managing inflammatory disorders because it has a demonstrated active redox action and thus acts as either a pro-oxidant or antioxidant agent in addition to its cytoprotective efficacy on cells under oxidative stress [8,15]. ATR revealed other interesting impacts on cancer cells due to its capacity to intercalate with DNA and prevent topoisomerase II enzyme, without affecting topoisomerase I [16]. Additionally, Shukla et al. [17] reported that ATR is a powerful inhibitor of some metabolic enzymes including ornithine decarboxylase (ODC), arginine decarboxylase, and arginase, that are associated with polyamine metabolisms.

Although ATR has been studied for their antiparasitic activity against several protozoan parasites, there have no reports on its antipiroplasmic efficacy. It is against this backdrop that the existing study intended to examine the growth-inhibitory efficacy of ATR as well as their combined effect with DA, AV, and clofazimine (CF) on *T. equi*, *B. divergens, B. bigemina*, *B. caballi,* and *B. bovis* multiplication in vitro. In addition to the investigation of its chemotherapy prospects against *B. microti*-infected mice as well as its cytotoxicity using three different mammalian cells (mouse embryonic fibroblast (NIH/3T3), human foreskin fibroblast (HFFs), and Madin-Darby bovine kidney (MDBK)), which are the major hosts of all tested parasites.

## 2. Results

### 2.1. The Inhibition Assay of ATR In Vitro

**Figure 2:**
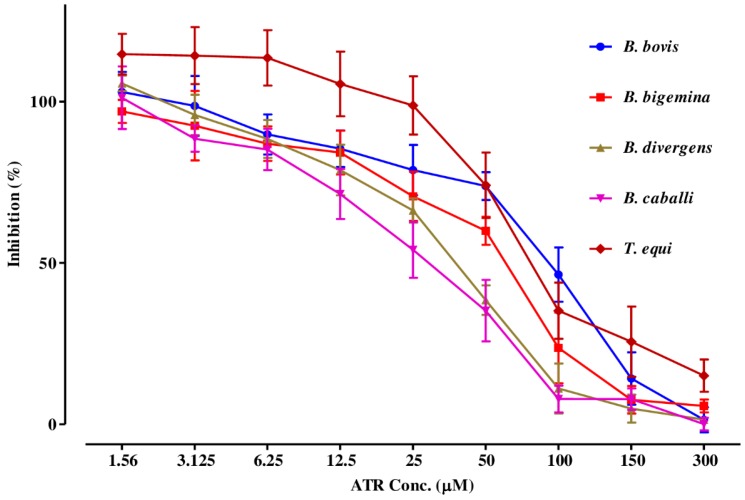
The relationship between the inhibition percentage and the concentrations of ATR (µM) on *T. equi*, *B. divergens*, *B. bigemina*, *B. caballi*, and *B. bovis*. The non-linear regression (curve fit analysis) in the GraphPad Prism software used for IC_50_ calculations. The percentage of parasite growth inhibitory efficacy is calculated as the percentage of parasites inhibited divided by that of the positive control wells and the result was subtracted from the negative control wells.

The preparatory assessment of ATR was performed to detect its efficacy on the host erythrocytes prior to the subculture of *T. equi* and *B. divergens*. The parasite proliferation did not show a significant difference between red blood cells (RBCs) treated with ATR and the untreated one for both parasites. Regarding the in vitro-inhibition efficacy, ATR affected all treated parasites multiplication in a dose-related manner (Figure 2).

ATR, DA, AV, and CF suppressed *T. equi, B. divergens, B. bigemina, B. caballi*, and *B. bovis* multiplication at IC_50_ values shown in Table 1.

Subsequently, the viability tests revealed that ATR at 2 × IC_50_ concentration completely suppressed *B. bigemina*, *B. caballi,* and *B. bovis* multiplication, while 4 × IC_50_ concentration cleared *B. divergens*. ATR at a concentration of 6 × IC_50_ suppressed the multiplication of *T. equi* (Table 2).

### 2.2. In Vitro Potential of the Combination of ATR with DA, CF, and AV

The ATR-DA combined treatment was additive toward *B. caballi,* while synergistic effect toward *T. equi, B. divergens*, *B. bigemina,* and *B. bovis*. The ATR-CF combined treatment was synergistic toward all tested parasites. The ATR-AV combined treatment was synergetic toward *B. caballi, B. divergens* and *B. bovis,* while revealed additive efficacy toward *T. equi* and *B. bigemina* (Table 3).

### 2.3. Toxicity of ATR on Normal Cell Lines

Cytotoxicity assay of ATR was assessed on HFFs, NIH/3T3, and MDBK cell lines (Table 1). ATR at a concentration of 1000 µM showed inhibition on NIH/3T3, HFFs and MDBK cells at EC_50_ values of 637.5 ± 12, 750 ± 15, and 775 ± 16 µM, respectively (Table 1). 

For ATR, the highest selectivity index (ratio of the effective concentration of ATR on the cell cultures to its inhibitory concentration on the parasites) was achieved on *T. equi* and found to be 10.9, 9 and 10.6 times toward NIH/3T3, HFFs, and MDBK cells, respectively (Table 4).

### 2.4. The In Vivo Chemotherapeutic Potential of ATR in Mice

**Figure 3:**
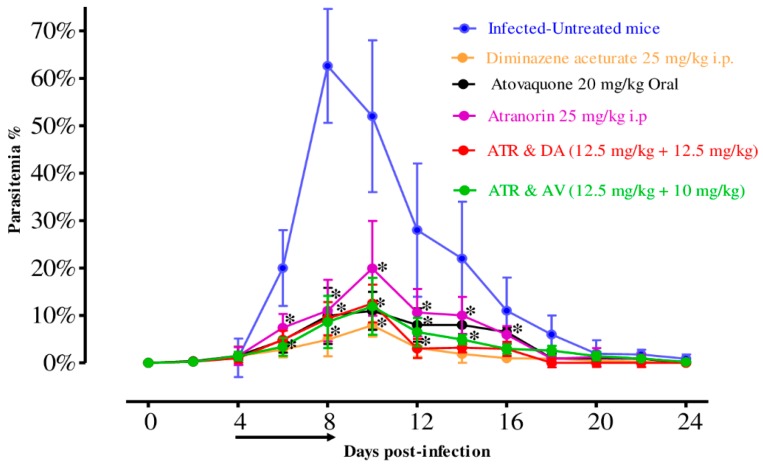
In vivo chemotherapeutical prospect of ATR on *B. microti* and the chemotherapeutic potential of DA-IP, AV-oral, ATR-IP, ATR-DA, and ATR-AV treatment when compared with the positive group. The arrow shows 5 successive days of drug administration starting from day 4 to 8 p.i. The asterisks (*) show the significant variation (*p* < 0.05) between drug-treated and positive groups. Parasitemia was detected using Giemsa-stained thin blood smears by counting infected RBCs (iRBCs) among 2000 RBCs.

To examine the in vivo chemotherapeutical potential of ATR, female BALB/c mice were infected by *B. microti* and ATR was administered either alone or in combinations. On the eighth day post-infection (p.i.), control mice treated with double distillate water (DDW) exhibited rapid growth of parasitemia reached 62.6% and the parasitemia reduced slowly on the subsequent days. In the ATR-treated group, the peak parasitemia level reached 19.92% on day 10, while it reached 7.94% and 10.99% on day 10 in DA at 25 mg/kg and AV at 20 mg/kg, respectively (Figure 3). According to microscopic examinations, parasitemia was not detected in groups treated with DA, AV, and ATR on days 16, 18 and 26 p.i., respectively. For the combination-treated groups, the levels of peak parasitemia exhibited 12.52% and 12.92% in 12.5 mg/kg body weight (BW) ATR + 12.5 mg/kg BW DA and 12.5 mg/kg BW ATR + 10 mg/kg BW AV on days 10 and 12, respectively (Figure 3). The first day on which parasitemia was not detected by microscopy was day 20 and 24 p.i. with ATR-DA and ATR-AV, respectively.

**Figure 4:**
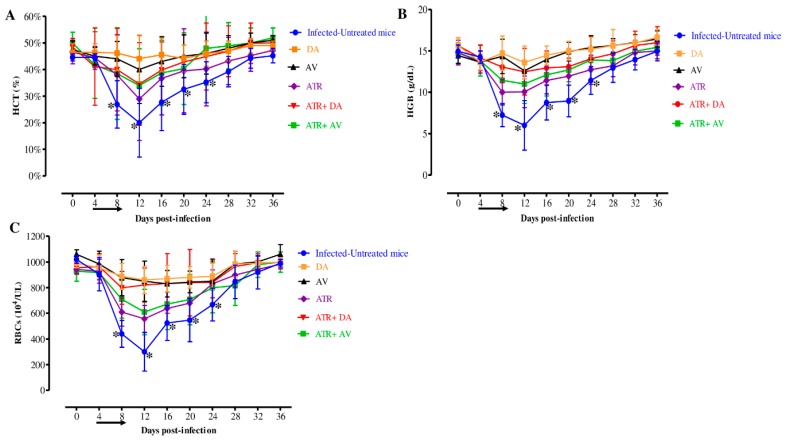
Hematology parameter changes in treated mice in vivo. Graphs showing the (**A**) hematocrit (HCT), (**B**) hemoglobin (HGB), and (**C**) red blood cells (RBCs) changes in treated mice compared to the infected-untreated mice. Asterisks (*) show significant variation (*p* < 0.05) between drug-treated and positive groups. The arrow shows five successive days of drug administration starting from day 4 to 8 p.i.

Furthermore, there are statistically significant variations (*p* < 0.05) in the hematocrit (HCT) percentage, hemoglobin (HGB) concentration, and RBCs count detected between the drug-treated groups and DDW group on the eighth, twelfth, sixteenth and twentieth days (Figure 4A–C).

**Figure 5:**
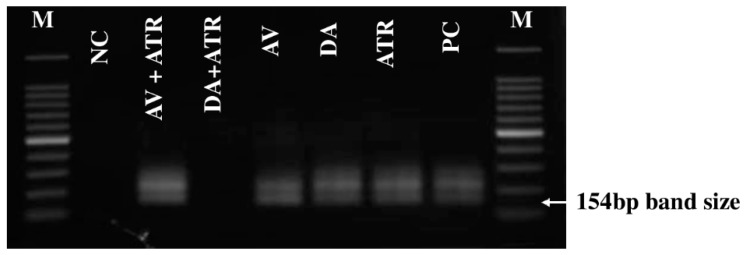
The molecular examination of *B. microti* in the blood of all treated groups on day 45. M refers to the marker, NC refers to the negative control group (untreated-uninfected), and PC refers to the positive control group (untreated-infected). The arrow indicates the 154 bp band length for *B. microti* positive cases.

PCR amplification detected the absence of parasite DNA in the ATR-DA group on day 45, while *B. microti* DNA was still noticed until day 45 in all other groups (Figure 5).

## 3. Discussion

This study revealed that ATR suppressed the in vitro multiplication of piroplasm parasites. The IC_50_ values obtained from ATR was lower than that showed by N-acetyl-L-cysteine [18], Allicin [19], thymoquinone against equine piroplasms parasites [20], norfloxacin, ofloxacin [21], *trans*-chalcone and chalcone hydrate against *B. divergens* [22], clodinafop-propargyl against bovine *Babesia* [23] and fusidic acid [24] and chalcone hydrate toward *B. bovis* [22] and ivermectin against *B. bigemina* and *T. equi* [25]. While its IC_50_ value was higher than that of ellagic acid [26], nitidine chloride and camptothecin [27] and 17-DMAG [28]. The ATR effectiveness against piroplasm parasites was compatible with the previous report, Susithra et al. [29] who showed that ATR has a potent anti-*Plasmodium* effect in vitro through inhibiting plasmepsin II (PLII) and dihydrofolate reductase (DHFR) malarial proteins. More so, ATR could be an attractive compound for developing new antiprotozoal molecules with potential inhibition of PLII and DHFR [11,12]. Although the existing data cannot explain how ATR can act against the tested species, therefore, additional research is urgently needed to elucidate its mechanism of action against piroplasm parasites.

The CCK test used to examine the cytotoxicity of ATR revealed its effect on NIH/3T3, HFFs, and MDBK cell viability with a slightly high selective index value. Meaning that ATR is more likely to affect the viability of piroplasm parasites rather than host cells. This finding conforms to the report by de Melo et al. [8] that ATR lacked toxicity in animal studies. In contrast, Zofou et al. [13] showed that ATR had mild cytotoxic action on LLC/MK2 monkey kidney epithelial cells. However, several reports have evaluated the cytotoxic activity of ATR against several cancers and normal cell cultures originated from human and animal cells, only a few cases documented the cytotoxicity of this compound against normal cells [30,31].

Nowadays, combination chemotherapies are being reported to alleviate serious diseases, including pulmonary tuberculosis, malignancy, immune deficiency syndrome, and some protozoal diseases to promote higher therapeutic efficacy [28]. Therefore, the present study examined the in vitro combination efficacy of ATR with three other drugs—AV, DA, and CF. These results indicate that the effects of ATR, when coupled with CF, AV, or DA were additive or synergistic against the five tested parasites. Interestingly, these results are compatible with Susithra et al. [29], in which they report that the ATR-2β,3β,19α-trihydroxyurs-12-en-28-oic acid combined treatment exhibited synergistic efficacy toward *Plasmodium species* with Combination Index equal 0.82, whereas its combined treatment with quinine revealed a slight antagonistic efficacy. Therefore, ATR and DA, AV, and CF combinations have prospects to be used as a treatment option of animal and human babesiosis.

The viability results demonstrated that ATR has the ability to restrict piroplasm parasites regrowth. For instance, ATR-treated *B. caballi*, *B. bigemina*, and *B. bovis* did not revive at 2 × IC_50_, whilst *B. divergens* did not revive at 4 × IC_50_ concentration. On the other hand, *T. equi* treated with ATR relapsed even at 6 × IC_50_ concentration. These results revealed the effectiveness of ATR toward *Babesia* parasites rather than *Theileria* parasites. The only possible explanation is that *T. equi* might behave through another mode of action to deal with stress-induced ATR therapy than that of *Babesia* species. These findings are compatible with previous studies, which explained that different parasites during infections try to use different defense mechanisms based on their environment [22,25,27]. However, the mechanism delay by which ATR could not completely kill *T. equi* parasite still unclear.

The in vitro inhibitory effects of ATR motivated us to assess their chemotherapeutical prospects on *B. microti* infection in mice, and we found that it was indeed effective in this context as well. The chemotherapeutic potential of ATR on the multiplication of *B. microti* was lower than those with DA and AV with no apparent adverse effects in mice. The i.p. administration of ATR exhibited chemotherapeutic effect higher than the 34%, 31%, 49%, 58.3%, 37%, and 49% shown by enoxacin, norfloxacin, and ofloxacin [21], allicin [19], thymoquinone [20], and ellagic acid [26], respectively. Whereas oral administration of HYD and DFMO exhibited a chemotherapeutic effect lower than the 89% and 91% shown by 17-DMAG [28] and nitidine chloride [27], respectively but similar to that shown by *trans*-chalcone [22]. These results are compatible with those ofde Melo et al. [8], who reported the anti-inflammatory activity of ATR, without any significant cytotoxicity in Wistar rats. Although DA is the most effective antibabesial drug used in the veterinary field, it has not been able to remove all parasites from the host animals. As a result, the disease can recur in treated animals. Moreover, restlessness, tissue injury at the site of injection, and abdominal pain were observed in animals after the DA treatment in addition to that it has not yet been approved for human use [32]. Therefore, a good combinatorial antibabesial drug is urgently needed. Interestingly, ATR–DA and ATR–AV combinations revealed higher chemotherapeutic efficacy when compared to that shown by ATR single-treatment, however it still less than that of DA or AV. Moreover, the PCR examination confirmed the disappearance of *B. microti* DNA from the ATR-DA combined group on day 42, emphasizing that ATR is a good combinatorial drug. Nevertheless, ATR, like DA and AV, prohibited anemia development in mice, although temporal reductions were observed in HCT, RBCs, and HGB. Furthermore, no obvious toxic signs or promoted anemia were observed in mice due to ATR treatment suggesting its safety for use in clinical trials. However, it is worth noting that additional studies should be conducted in the near future to verify how ATR can act against piroplasm parasites to open the way to understanding the effectiveness of its interaction with existing antibabesial drugs.

Interestingly, Gaikwad et al. [33] showed that ATR enhances the production of probiotic bacteria at a low pH, by increasing the animal growth performance. In addition, the immunomodulatory effect of ATR has been shown by measuring its efficacy on both human-isolated polymorphonuclear leukocytes (PMNs) and the respiratory eruption of whole blood phagocytes by detecting the intra- and/ or extra-cellular ROS, using luminol- or lucigenin-based chemiluminescence [10]. Such medicinal characteristics are important for piroplasmosis treatment because piroplasmosis infection is not only correlated with emaciation and poor growth performance in cattle but also with immunosuppression [34].

## 4. Materials and Methods

### 4.1. Chemical Reagents

Stock solutions (10 mM) in dimethyl sulfoxide (DMSO) of ATR, CF, AV (Sigma-Aldrich, Tokyo, Japan) and DA (Novartis Animal Health, Tokyo, Japan) were stored at −30 °C and used for antibabesiosis evaluation. Reference drugs including DA, CF, and AV were used either individually or combined with ATR for both the in vivo and in vitro studies. For the fluorescence test, SYBR Green I (SGI) stain (10,000×, Lonza, Alpharetta, GA, USA) was mixed with the lysis buffer containing saponin (0.016% *w/v*), EDTA (10 mM), Triton X–100 (1.6% *v/v*), and Tris (130 mM at pH 7.5) which was filtered using a polyethersulfone (0.22 µm) and kept at 4 °C.

### 4.2. Ethical Approval

The in vivo experiments were carried out in conformity with the local guidelines for animal experiments, as approved by the Obihiro University of Agriculture and Veterinary Medicine, Japan (animal experiment accession number: 29-016-8).

### 4.3. Cultivation Condition In Vitro

#### 4.3.1. The Parasites Cultivation In Vitro

*Babesia* parasite was incubated and maintained at 37 °C in a humidifying chamber under 5% CO_2_, 5% O_2_, and 90% N_2_ atmosphere using a microaerophilic stationary-phase culture for conducting the in vitro experiment [35,36,37]. Briefly, Germany strain *Babesia divergens* was cultured in cattle RBCs in Roswell Park Memorial Institute 1640 (RPMI 1640; Sigma-Aldrich, St. Louis, MO, USA) medium replenished with 40% cattle serum, while culture medium 199 (M199; Sigma-Aldrich) was utilized for the Argentina strain *B. bigemina* and Texas strain *B. bovis*, and *T. equi* USDA strain cultured in cattle RBCs supplemented with 40% cattle serum and horse RBCs maintained in hypoxanthine (MP Biomedicals, Santa Ana, CA, USA; final concentration 13.6 μg/mL) and 40% horse serum, respectively. GIT medium complemented with 40% horse serum was used as a growth medium for the USDA strain of *B. caballi* cultured in horse RBCs. To ensure free-bacterial contamination, amphotericin B (0.15 μg/mL) (Sigma-Aldrich), streptomycin (60 U/mL) and penicillin G (60 U/mL) were added to all medium.

#### 4.3.2. Evaluation of the Impacts of ATR on RBCs of Bovines and Equines

Prior to parasite subculture, a concentration of 400 µM of ATR was mixed with fresh bovine and equine RBCs and incubated at a humidified incubator for 3 and 6 h [27,37]. Afterward, the pretreated-RBCs were mixed with *B. divergens* and *T. equi* infected RBCs (iRBCs) after washing thrice with PBS to achieve 1% parasitemia. Thereafter, using a 24-well plate, an aliquot of iRBCs (100 μL) was mixed with culture media (900 μL); the control RBCs were left untreated. To monitor the parasitemia and any side effects due to the pretreatment, Giemsa-stained smears were prepared every 24 h for four days.

#### 4.3.3. The In Vitro Growth Inhibitory Effects of ATR and Its Combination Efficacy with DA, CF, and AV

The *Babesia* fluorescent assay was carried out on the in vitro culture as previously reported elsewhere [28,37,38] to assess the growth of the inhibitory effectiveness of ATR and the combination treatment with DA, CF, and AV. Briefly, in three separate trials, using two-fold dilution, different concentrations of ATR, DA, CF, and AV were prepared in the culture medium and added in 96-well plates in triplicate with 1% parasitemia for *T. equi*, *B. divergens* and *B. caballi* at 5% HCT, while for *B. bigemina* and *B. bovis* using 2.5% HCT. The drug combined efficacy was conducted at the same time with the single-treatment assay in accordance with the Chou–Talalay method [36,37,39]. Five selected dilutions (0.25×, 0.5×, 1×, 2× and 4× the IC_50_) of ATR with CF or DA or AV (Supplementary Table S1) were set up in three sets of duplicate wells and the parasite cultures were incubated for four consecutive days at 37 °C humidifying incubator in 90% N_2_, 5% O_2_, and 5% CO_2_ atmosphere without changing medium. On day four of culture, an aliquot (100 µL) of lysis buffer mixed with 0.2μL/mL SG1 was added per well; subsequently, it was covered with aluminum foil to prevent exposure to light. After incubation for 6-h at 37 °C, fluorescence readings were acquired on a spectrofluorimeter (Fluoroskan Ascent, Thermo Fisher Scientific, Oceanside, CA, USA) with a 485 nm excitation wavelength and a 518 nm emission wavelength.

#### 4.3.4. Parasite Viability Test In Vitro

The viability studies of ATR-treated parasites were monitored via microscopy as reported elsewhere [25,26,37]. In a 96-well microtiter plate, an aliquot of 1% parasitemia (10 µL) of iRBCs was cultivated with 90 µL of respective media containing various concentrations of ATR and DA for 4 successive days with changing media daily. The concentrations used in this experiment were 0.25×, 0.5×, 1×, 2×, 4×, and 6× the IC_50_. On the fifth day, a mixture of iRBCs (3 µL) from each well and fresh equine or bovine RBCs (7 µL) was transferred to another plate, cultured in a medium free from drug and then left for an additional six days. The total parasite clearance was recorded as negative, while the relapse of parasites was recorded as positive.

### 4.4. Cytotoxicity Assay

#### 4.4.1. Cell Cultures

Cultures of human foreskin fibroblast (HFF; HFF-1 ATCC^®^ SCRC-1041™), Madin–Darby bovine kidney (MDBK; ECACC) and mouse embryonic fibroblast (NIH/3T3; ATCC^®^ CRL-1658™) cells were retrieved from −80 °C stock and cultured continuously at 37 °C under atmosphere 5% CO_2_ in our laboratory. The NIH/3T3 and HFFs cells were maintained in Dulbecco Modified Eagle’s Medium (DMEM; Gibco, Grand Island, NY, USA), while MDBK cell line grown in Minimum Essential Medium Eagle (MEM; Gibco). Each medium was treated with 0.5% penicillin/streptomycin (Gibco), 10% inactivated fetal bovine serum and 2 mM glutamine. Every 72 to 96 h, the medium was replaced, and once 80% confluence was reached, the cell collection was performed by sub-culture protocol. To confirm the absence of mycoplasma contamination, 4,6-diamidino-2-phenylindole dihydrochloride stain (Sigma-Aldrich) was used.

#### 4.4.2. Cytotoxic Action of ATR, DA, CF, and AV on Normal Cells

The cell viability test was conducted in a 96-well plate as described elsewhere [25,37,38]. Briefly, an aliquot of (100 µL) cells was implanted at a concentration of 5 × 10^4^ cells/mL in DMEM or MEM with fetal bovine serum and incubated overnight under atmosphere 5% CO_2_ at 37 °C for attachment. Using two-fold dilutions, aliquots (10 µL) of drugs were added in triplicate per well to attain final concentrations of 50 to 1000 µM and further incubated for 24 h. The positive control wells containing cells mixed with the medium in 0.4% DMSO, whereas the negative control wells containing culture medium only. After a 24-h incubation, Cell Counting Kits-8 (CCK-8, 10 µL) was added to each well and then plate incubation was conducted for an additional 3 h and a microplate reader was used to assess the absorbance at 450 nm.

### 4.5. In Vivo Experiments

#### 4.5.1. Chemotherapeutic Effects of ATR against *B. Microti*

ATR was examined for its in vivo chemotherapeutic effectiveness using *B. microti*–infected BALB/c mice according to a procedure described elsewhere [25,27,36]. Briefly, 35 female eight-week-old mice were placed in an environment free from pathogens with 22 °C temperature and adjusted humidity and under 12 h light and 12 h darkness and randomly distributed into seven groups. The mice in groups 2 through 7 obtained 500 µL of 1 × 10^7^ *B. microti* iRBC by intraperitoneal (i.p.) injection. Group 1 served as a negative control and was neither infected nor treated. At 1% parasitemia, drug treatment of the mice by i.p. or oral routes started, continuing for five days. Group 2 act as a positive control group and received 95% DDW and 5% DMSO. Groups 3 and 4 were served as the reference drug controls and received 25 mg/kg BW of DA and 20 mg/kg BW of AV. Group 5 obtained an i.p. injection of 25 mg/kg of ATR, respectively, whilst sixth and seventh groups administered combinations of 12.5 mg/kg BW ATR + 12.5 mg/kg BW DA and 12.5 mg/kg BW ATR + 10 mg/kg BW AV, respectively, by intraperitoneal and oral routes. The drug administration lasted for five days starting from the fourth day to the eighth day p.i., and parasitemia was checked by preparing Giemsa-stained smears every two days in about 2000 RBCs by microscopy until day 45 p.i. Furthermore, the hematological parameters, including hemoglobin (HGB), RBCs, and HCT, were determined by an automatic hematology analyzer (Celltac α MEK-6450, Nihon Kohden, Tokyo, Japan) every four days. At the end of the in vivo experiment, an anesthetic system using an inhaler containing isoflurane was used to euthanize all mice by placing them in the induction chamber, adjusting the oxygen flowmeter to 0.8 to 1.5 L/min and vaporizer to 3% to 5%. When mice were completely anesthetized, all of them were killed by cervical dislocation and the blood was gathered by cardiac puncture for PCR detection of *B. microti* DNA.

#### 4.5.2. PCR Identification of *B. Microti* DNA Extracted from All Treated Groups

The PCR amplification was used to detect the ability of drugs to completely clear the parasites from the blood through confirming the parasite DNA disappearance from all treated groups. At the end of the in vivo experiment, all mice were euthanized and a QIAamp DNA Blood Mini Kit (Qiagen, Tokyo, Japan) was used to extract *B. microti* DNA from the blood samples collected on day 45 p.i. from all groups. PCR cycling was conducted as previously reported [25,26]. Briefly, PCR amplifications were performed in a 10 µL reaction mixture containing 0.5 µM of each primer, 0.1 µL of Platinum SuperFi™ DNA polymerase (Thermo Fisher Scientific, Tokyo, Japan), 0.2 mM dNTP mix, 2 µL of 5× SuperFi ™ buffer, 4.9 µL of DDW and 1 µL of DNA template. The cycling conditions were: 94 °C for 30 s, 53 °C for 30 s and 72 °C for 30 s as denaturation, annealing and extension steps for 35 cycles, respectively, using the forward (5′-CTT AGT ATA AGC TTT TAT ACA GC-3′) and reverse (5′-ATA GGT CAG AAA CTT GAA TGA TAC A-3′) primers. Afterward, 1 µL of DNA template from the first PCR amplification was used as the template for the nPCR assays under the same cycling conditions, using the forward (5′-GTT ATA GTT TAT TTG ATG TTC GTT T-3′) and reverse (5′-AAG CCA TGC GAT TCG CTA AT-3′) primers. The PCR products were stained with ethidium bromide and visualized under the UV transilluminator after a resolution by electrophoresis in 1.5% agarose gel and the bands were considered positive at 154 bp.

### 4.6. Statistical Analysis

The IC_50_ values of ATR, DA, CF, and AV were established from the in vitro growth inhibition by nonlinear regression curve fit on a GraphPad Prism (GraphPad Software Inc., San Diego, CA, USA). CompuSyn software was used for combination index (CI) values calculation and the synergetic degree was established as the average weighted CI values by using the following formulae; ((1 × IC_50_) + (2 × IC_75_) + (3 × IC_90_) + (4 × IC_95_))/10 and the resulted values were demonstrated using the recommended CI scale; lower than 0.90 was considered synergetic, between 0.90–1.10 was considered additive, while higher than 1.10 was considered antagonistic developed previously [39,40]. The significant variations (*p* < 0.05) among group mean values on parasitemia and one-way ANOVA Tukey’s test in GraphPad Prism version 5.0 was used to analyze hematology profiles in mice infected with *B. microti*.

## 5. Conclusions

The findings in our study support the multidirectional biological activities of ATR. ATR showed potential anti-piroplasmic activity against several piroplasm parasites in vitro and in vivo. The ATR + DA, ATR + AV, as well as ATR + CF combinations, were either synergistic or additive toward all tested piroplasm parasites, which implies that ATR is an interesting lichen substance that might possess a potential value for treating clinic diseases in animals and humans either alone or in combination with other drugs. Therefore, it could open the way for the search for other β-orcinol and lichen acid analogs with greater therapeutic efficacy and minimal toxic activity. However, further investigations involving clinical trials on infected cattle and humans may be necessary and further evaluating the efficacy of other lichen acid derivatives against piroplasm parasites.

## Figures and Tables

**Table 1:** IC_50_ values of ATR, DA, AV, and CF.

Parasite	IC_50_ (µM) ^a^			
	ATR	DA	AV	CF
*B. bovis*	98.4 ± 4.2	0.35 ± 0.06	0.04 ± 0.00	8.24 ± 1.7
*B. bigemina*	64.5 ± 3.9	0.68 ± 0.09	0.70 ± 0.04	5.73 ± 1.9
*B. divergens*	45.2 ± 5.9	0.43 ± 0.05	0.04 ± 0.00	13.8 ± 4.3
*B. caballi*	46.6 ± 2.5	0.02 ± 0.00	0.10 ± 0.01	7.95 ± 1.8
*T. equi*	71.3 ± 2.7	0.71 ± 0.05	0.09 ± 0.06	2.88 ± 0.9

^a^ IC_50_ values of ATR, DA, AV, and CF on all tested parasites in vitro. The dose-response curve using nonlinear regression (curve fit analysis) was used to detect all of these values. The values obtained from the means of triplicate experiments.

**Table 2:** Viability of parasites treated with ATR.

Parasite	ATR			Negative Control
	Concentration (µM) ^a^		Viability	
*B. bovis*	A_1_	24.6	+	
	A_2_	49.2	+	
	A_3_	98.4	+	+
	A_4_	196.8	–	
	A_5_	393.6	–	
	A_6_	590.4	–	
*B. bigemina*	A_1_	16.1	+	
	A_2_	32.25	+	
	A_3_	64.5	+	+
	A_4_	129	–	
	A_5_	258	–	
	A_6_	387	–	
*B. divergens*	A_1_	11.3	+	
	A_2_	22.6	+	
	A_3_	45.2	+	+
	A_4_	90.4	+	
	A_5_	180.8	–	
	A_6_	271.2	–	
*B. caballi*	A_1_	11.65	+	
	A_2_	23.3	+	
	A_3_	46.6	+	+
	A_4_	93.2	–	
	A_5_	186.4	–	
	A_6_	279.6	–	
*T. equi*	A_1_	17.8	+	
	A_2_	35.6	+	
	A_3_	71.3	+	+
	A_4_	142.6	+	
	A_5_	285.2	+	
	A_6_	427.8	–	

^a^ A_1_–A_6_ refers to 0.25×, 0.5×, 1×, 2×, 4×, and 6× the IC_50_ of ATR. Results are calculated as the mean values from three separate trials ± SD, a positive (+) indicates parasites regrowth, and a negative (–) shows the parasites total clearance after drug pressure withdrawal using microscopy assay.

**Table 3:** Combination effect of ATR with DA, AV, and CF in vitro.

Parasite	Drug Combination ^a^	CI Values				Weighted Average CI Values ^b^	Degree of Association ^c^
		IC_50_	IC_75_	IC_90_	IC_95_		
*B. bovis*	ATR + DA	1.008	0.973	0.702	0.778	0.817	Synergism
	ATR + AV	0.700	0.625	0.882	0.826	0.79	Synergism
	ATR + CF	0.783	0.398	0.892	0.919	0.793	Synergism
*B. bigemina*	ATR + DA	0.806	0.994	0.933	0.938	0.937	Synergism
	ATR + AV	0.903	1.007	1.003	1.91	1.356	Additive
	ATR + CF	0.719	0.382	0.181	0.393	0.359	Synergism
*B. divergens*	ATR + DA	0.774	0.957	0.879	0.807	0.855	Synergism
	ATR + AV	0.822	0.972	0.938	0.632	0.81	Synergism
	ATR + CF	0.922	0.791	0.772	0.883	0.835	Synergism
*B. caballi*	ATR + DA	1.627	1.098	0.795	0.968	1.008	Additive
	ATR + AV	0.482	0.052	0.372	0.572	0.399	Synergism
	ATR + CF	0.793	0.892	0.793	0.872	0.844	Synergism
*T. equi*	ATR + DA	0.881	0.692	0.999	0.952	0.907	Synergism
	ATR + AV	1.036	1.023	1.002	1.013	1.012	Additive
	ATR + CF	0.830	0.896	0.981	0.893	0.913	Synergism

^a^ Two-drug combination between ATR with DA, AV and CF at 0.25 × IC_50_, 0.5 × IC_50_, IC_50_, 2 × IC_50_, and 4 × IC_50_ (constant ratio) concentration. ^b^ The mean weighted CI value was estimated with the formula [(1 × IC_50_) + (2 × IC_75_) + (3 × IC_90_) + (4 × IC_95_)]/10. ^c^ The resulted values were demonstrated using the recommended CI scale; lower than 0.90 was considered synergetic, between 0.90–1.10 was considered additive, while higher than 1.10 was considered antagonistic. CI, combination index value; IC_50_, 50% inhibition concentration; ATR, atranorin; DA, diminazene aceturate; CF, clofazimine; AV, atovaquone.

**Table 4:** Selective index values of ATR, DA, AV, and CF.

Drug	Parasite	EC_50_ (µM) ^a^			Selective Index ^b^		
		MDBK	NIH/3T3	HFFs	MDBK	NIH/3T3	HFFs
**ATR**	*B. bovis*	775 ± 16	637.5 ± 12	750 ± 15	7.9	6.5	7.6
	*B. bigemina*	775 ± 16	637.5 ± 12	750 ± 15	12.0	9.9	11.6
	*B. divergens*	775 ± 16	637.5 ± 12	750 ± 15	17.1	14.0	16.6
	*B. caballi*	775 ± 16	637.5 ± 12	750 ± 15	16.6	13.7	16.1
	*T. equi*	775 ± 16	637.5 ± 12	750 ± 15	10.9	9	10.6
**DA**	*B. bovis*	˃100	˃100	˃100	˃285.7	˃285.7	˃285.7
	*B. bigemina*	˃100	˃100	˃100	˃147.1	˃147.1	˃147.1
	*B. divergens*	˃100	˃100	˃100	˃232.5	˃232.5	˃232.5
	*B. caballi*	˃100	˃100	˃100	˃5000	˃5000	˃5000
	*T. equi*	˃100	˃100	˃100	˃140.8	˃140.8	˃140.8
**AV**	*B. bovis*	˃100	˃100	˃100	˃2564	˃2564	˃2564
	*B. bigemina*	˃100	˃100	˃100	˃142.7	˃142.7	˃142.7
	*B. divergens*	˃100	˃100	˃100	˃2631	˃2631	˃2631
	*B. caballi*	˃100	˃100	˃100	˃980.4	˃980.4	˃980.4
	*T. equi*	˃100	˃100	˃100	˃1052	˃1052	˃1052
**CF**	*B. bovis*	34.7 ± 3.4	˃100	˃100	4.2	˃12.1	˃12.1
	*B. bigemina*	34.7 ± 3.4	˃100	˃100	6.1	˃17.5	˃17.5
	*B. divergens*	34.7 ± 3.4	˃100	˃100	2.5	˃7.2	˃7.2
	*B. caballi*	34.7 ± 3.4	˃100	˃100	4.4	˃12.6	˃12.6
	*T. equi*	34.7 ± 3.4	˃100	˃100	12.1	˃34.7	˃34.7

^a^ EC_50_ values of ATR, DA, AV, and CF on the tested cell lines. The dose-response curve using nonlinear regression (curve fit analysis) was used to detect all of these values. The values obtained from the means of triplicate experiments. ^b^ Selective index calculated as the ratio between the concentration that causes safety problems in cell lines and the concentration that is used for efficacy in each parasite. MDBK, Madin-Darby bovine kidney; NIH/3T3, Mouse embryonic fibroblast; HFF, Human foreskin fibroblast.

## Supplementary Materials

The following materials are available online at https://www.mdpi.com/2076-0817/9/2/127/s1, Table S1: Concentrations of the in vitro combined treatment of atranorin (ATR) with clofazimine (CF), atovaquone (AV) and diminazene aceturate (DA) against *Babesia* and *Theileria* parasites. Original data of hematocrit (HCT) percentage, hemoglobin (HGB) concentration, and RBCs count.
